# Urinary excretion of thyroid hormone in CKD patients: a proof-of-concept of nephrogenic hypothyroidism

**DOI:** 10.1080/0886022X.2023.2293224

**Published:** 2023-12-12

**Authors:** Rena Yuasa, Masaki Muramatsu, Akinobu Saito, Hiroyoshi Osuka, Toshisuke Morita, Yuko Hamasaki, Ken Sakai

**Affiliations:** a Department of Clinical Medicine, Toho University Faculty of Nursing, Tokyo, Japan; b Department of Nephrology, Toho University Faculty of Medicine, Tokyo, Japan; c Department of Clinical Laboratory, Toho University Omori Medical Center, Tokyo, Japan; d Department of Laboratory Medicine, Toho University Faculty of Medicine, Tokyo, Japan

**Keywords:** CKD, hypothyroidism, nephrogenic hypothyroidism, proteinuria, urinary excretion of thyroid hormones, independent of autoimmunity, thyroid hormone-binding proteins

## Abstract

**Purpose:**

Patients with chronic kidney disease (CKD) complicated by hypothyroidism exhibit a higher prevalence of urine protein than that in the general population. This study was aimed at investigating thyroid hormones and thyroid hormone-binding proteins excreted in urine to elucidate the urine protein-associated underlying mechanisms of hypothyroidism.

**Methods:**

Between November 2016 and August 2018, thyroid function (serum free T3 [sFT3], free T4 [sFT4], and thyroid-stimulating hormone [sTSH]), kidney function (estimated glomerular filtration rate [eGFR]), thyroid antibodies and albumin (Alb) were evaluated in 99 Japanese CKD patients with proteinuria at our outpatient clinic. A urine examination was also performed to assess the following parameters: total T3, total T4, TSH, Alb, preAlb, thyroid-binding globulin, and protein.

**Results:**

The median patient age at study recruitment was 60 years; 50 patients (50.5%) were male. The median eGFR and Alb level were 20.3 ml/min/1.73 m^2^ and 3.8 g/dL, respectively. 21 patients (21.2%) were diagnosed with nephrotic syndrome (NS). The median sFT3, sFT4, and sTSH levels were within normal limits. Approximately 70% of the patients had thyroid dysfunction and 51.5% had overt or subclinical hypothyroidism without predominantly antibody positive. Regarding NS and non-NS patients, age and Alb were significantly different between these groups, while sex and eGFR were not significant, but the urinary T4 and TSH levels were higher in the NS group; thus, more severe hypothyroid.

**Conclusion:**

We found a significant association between hypothyroidism and NS regardless of sex and antibodies. Urinary loss of thyroid hormones must be a factor influencing hypothyroidism independent of autoimmunity.

## Introduction

On the basis of our clinical experience, we concluded that many clinical manifestations of chronic kidney disease (CKD) are similar to those of hypothyroidism. We often encounter symptoms such as general fatigue, edema, and shortness of breath, which are typical of both conditions. Thus, hypothyroidism may be masked in patients with CKD. We believe this to be one of the reasons why hypothyroidism in patients with CKD might go unnoticed, although it may require suitable treatment. This situation is clinically concerning and should be promptly addressed in daily medical practice.

In a previous study, we found that CKD complicated by hypothyroidism is highly prevalent [1]. Age, estimated glomerular filtration rate (eGFR), and urine protein (UP) and serum albumin (Alb) levels were markedly related to the prevalence of hypothyroidism, whereas sex was not, which was surprisingly contradictory to the prevalence of hypothyroidism in the general population [2]. Several researchers have investigated the relationship between hypothyroidism and CKD [3–8]; however, no definite analysis or assessment has been conducted. Moreover, among malnourished patients, such as those with CKD, a reduction in the total binding protein level led to a reduction in the total T4 and total T3 levels. This might have led to a misdiagnosis of authentic hypothyroidism and non-thyroidal illness syndromes (NTI) such as low T3 and low T4 syndromes [9–12]. Also, several researchers have recognized the relationship between nephrotic syndrome and hypothyroidism. In 1991, Fonseca reported four nephrotic syndrome patients with hypothyroidism without thyroid antibodies [13]. Bevenga advocated the dose of thyroid hormone replacement therapy must be increased in nephrotic syndrome because of urinary loss of free and protein-bound thyroid hormones [14].

This study was thus aimed at elucidating the mechanisms causing hypothyroidism in CKD patients with proteinuria, focusing on the loss of thyroid hormones in the urine, in order to prove the concept of nephrogenic hypothyroidism.

## Materials and methods

### Study design and setting

**Figure 1.**
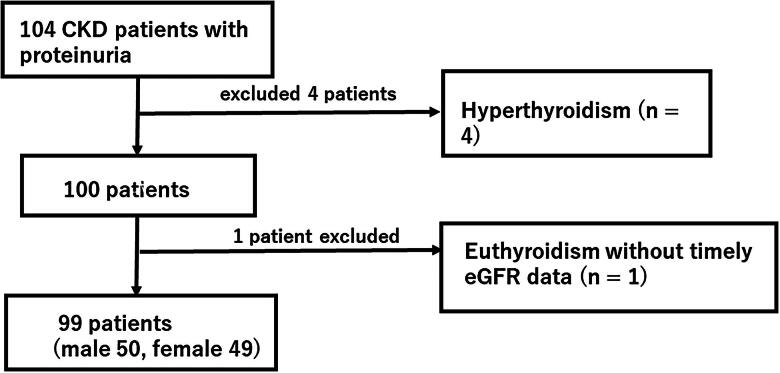
Flow diagram of patient selection. Abbreviations: CKD: chronic kidney disease; eGFR: estimated glomerular flow rate.

This cross-sectional study recruited Japanese CKD patients with proteinuria based on clinical examinations at a single center. Patients were admitted to our renal clinic outpatient clinic between November 2016 and August 2018. Initially, the total number of CKD patients with proteinuria was 104; however, four patients who were under treatment for hyperthyroidism were excluded. One hundred patients consented to undergoing tests for the measurement of the following parameters: thyroid function (serum free T3 [sFT3], free T4 [sFT4], and thyroid-stimulating hormone [sTSH]), kidney function (eGFR), and Alb. In addition, urinary levels of the following markers were determined: total T3 (UT3), total T4 (UT4), TSH (UTSH), Alb (UAlb), preAlb (UpreAlb), thyroid-binding globulin (UTBG), and protein. Since one patient’s eGFR data was absent, and CKD stage could not be determined, the remaining 99 patients (Figure 1) were included in the analysis.

### Laboratory measurements

Patient samples were examined in our hospital’s laboratory, and the following parameters were measured: eGFR; Alb; serum and urinal levels of TSH, FT3, and FT4; and levels of UP, UAlb, UpreAlb, UTBG, UT3, UT4, and UTSH. Thyroid hormone levels were determined using an electrochemiluminescence immunoassay (Roche Diagnostics K.K. Tokyo, Japan). Additionally, serum anti-thyroid peroxidase antibody (TPOAb) and anti-thyroglobulin antibody (TgAb) were measured. TPOAb and TgAb concentration were determined by electro-chemiluminescence immunoassay (ECLIA, Roche Diagnostics K.K. Tokyo, Japan). Thyroid hormone-binding proteins such as Alb, preAlb, and TBG for radioimmunoassay were obtained from LSI Medience Corporation (Chiyoda-ku, Tokyo, Japan). The reference ranges for sFT3, sFT4, sTSH, TPOAb, and TgAb were 2.26–4.15 pg/ml, 1.01–1.67 ng/dl, 0.32–4.12 mIU/ml, <16 IU/ml, and <28 IU/ml, respectively. There are no reference ranges for the urine components.

### Assessment of kidney function and thyroid function

The Japanese eGFR formula is as follows: [eGFR (ml/min/1.73 m^2^)] = 194 × Cr^−1.094^ × Age^−0.287^ (× 0.739 for female patients), where Cr stands for creatinine [15]. CKD was classified into five stages based on eGFR and the UP level in accordance with the 2009 Japanese Society of Nephrology evidence-based practice guidelines for the treatment of CKD [16]. eGFR was divided into the following six categories: G1, ≥90 mL/min/1.73 m^2^; G2, 60–89 mL/min/1.73 m^2^; G3a, 45–59 mL/min/1.73 m^2^; G3b, 30–44 mL/min/1.73 m^2^; G4, 15–29 mL/min/1.73 m^2^; and G5, <15 mL/min/1.73 m^2^. Proteinuria was divided into the following three categories: A1, <0.15 g/day; A2, 0.15–0.49 g/day; and A3, ≥0.50 g/day [17]. The amount of UP per day was converted into grams per gram of Cr (g/g Cr).

Nephrotic syndrome (NS) was diagnosed based on the Evidence-based Clinical Practice Guidelines for Nephrotic Syndrome 2020 [18]. The clinical definitions of NS are as follows: (1) Proteinuria: ≥3.5 g/day and continuous (comparable to ≥3.5 g/gCr at spot urine), (2) Hypoalbuminemia: serum Alb of ≤3.0 g/dL Serum total protein ≤ 6.0 g/dL is helpful.

Thyroid function was evaluated based on sTSH, sFT3, and sFT4 levels and categorized into five different condition (Table 1): overt hypothyroidism (OH), defined as low FT4 (<1.01 ng/dl) with elevated sTSH (>4.12 mIU/ml); subclinical hypothyroidism (SH), defined as elevated sTSH (>4.12 mIU/ml) with normal sFT4; low T3 syndrome (LT3S), defined as low sFT3 with normal sFT4 and sTSH; and low T4 syndrome (LT4S), defined as low FT4 and low and/or normal FT3 with normal TSH. Findings other than these four thyroid conditions were regarded as indicative of normal thyroid function (euthyroidism).

**Table 1.** Classification of thyroid function.

Thyroid function	FT3	FT4	TSH
Overt hypothyroidism (OH)	↓	↓	↑
Subclinical hypothyroidism (SH)	→	→	↑
LowT3 syndrome (LT3S)	↓	→	→
LowT4 syndrome (LT4S)	↓/→	↓	→

Abbreviations: FT3: serum free T3; FT4: serum free T4.

Reference ranges of FT3, FT4, and TSH were 2.26–4.15 pg/ml, 1.01–1.67 ng/dl, and 0.32–4.12 mIU/ml, respectively.

## Covariates

Covariates of age and sex were assessed based on the self-reporting by each patient.

### Statistical analysis

Statistical analysis was performed using JMP 16 software (SAS Institute, Inc., Cary, NC, USA). Continuous variables are expressed as medians (ranges), and categorical variables are expressed as numbers (percentages). Statistical significance for the two groups was assessed using the Steel-Dwass’s test for multivariate analysis.; the Pearson χ^2^ test was used to assess categorical variables. Differences with *p* < 0.05 were considered as statistically significant.

## Results

### Patient characteristics

The baseline patient characteristics and clinical and biochemical data of 99 CKD patients with proteinuria are shown in Table 2a. Twenty-one (21.2%) patients were diagnosed with NS. The median sFT3, sFT4, and sTSH levels were within normal ranges (Table 2b). Each factor exhibited a nonnormal distribution. The patients were classified as follows according to their CKD stages: 1-5. They were also classified into five groups according to their thyroid function: OH, 19.8%; SH, 21.8%; LT3S, 16.8%; LT4S, 9.9%; and normal, 31.7%. Thus, of the 99 patients, approximately 70% had thyroid dysfunction; especially, 41.6% had hypothyroidism including OH and SH.

**Table 2a.** Patient characteristics.

Parameter	Median (IQR or %)
Age	60 (45–73)
Sex male, n (%)	50 (50.5%)
eGFR ml/mon/1.73m2	20.3 (9.6–42.4)
Alb g/dl	3.8 (2.9–4.1)
CKD stage	G1 (3%)
	G2 (13%)
	G3 (25%)
	G4 (13%)
	G5 (46%)
Nephrotic, n (%)	21 (21.2%)
Underlying disease, *n* (%)	Chronic glomerulonephritis 23 (23.2%)
	Diabetic nephropathy 14 (14.1%)
	Nephrosclerosis 13 (13.1%)
	Tibulointerstitial nephritis 8 (8.1%)
	Congenital disease 6 (6.1%)
	Hereditary disease 3 (3.0%)
	Vasculitis 2 (2.0%)
	Unknown 31 (31.3%)
Thyroid function, n (%)	Overt hypothyroidism 31 (31.3%)
	Subclinical hypothyroidism 20 (20.2%)
	Low T3 syndrome 22 (22.2%)
	Low T4 syndrome 16 (16.2%)
	Normal 10 (10.1%)

Abbreviations: eGFR: estimated glomerular filtration rate; CKD: chronic kidney disease; IQR: interquartile range.

Reference ranges; eGFR > 60 mL/min/1.73m^2^, Alb≧3.9g/dl.

### Frequency distribution

**Figure 2.**
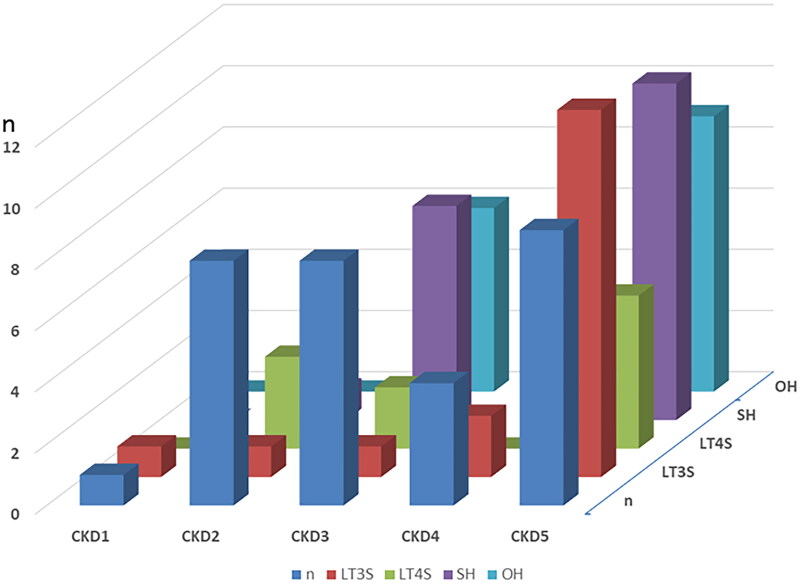
Association between CKD stage and thyroid function. Abbreviations: CKD: chronic kidney disease; n: normal; SH: subclinical hypothyroidism; OH: overt hypothyroidism; LT3S: low T3 syndrome; LT4S: low T4 syndrome;

**Figure 3.**
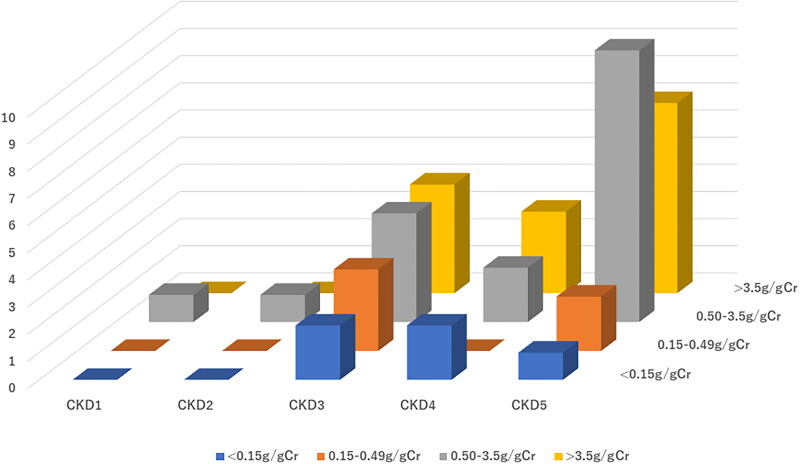
Association among CKD stage, up level, and hypothyroidism (OH + SH) (*n* = 42). Abbreviations: CKD: chronic kidney disease; SH: subclinical hypothyroidism; UP: urine protein; OH: overt hypothyroidism

The frequency distributions showing the associations between CKD stage and thyroid function (OH, SH, and LT3S and LT4S) are shown in Figure 2. Figure 2 showed that the prevalence of thyroid dysfunction was greater among patients with severe CKD with a high UP level; we evaluated this using a 3D graph. Figure 3 shows the association of CKD stage, UP level, and hypothyroidism (OH and SH). To focus only on SH and OH, a total of 42 CKD patients were analyzed without LT3S and LT4S. A high UP level at an advanced stage of CKD was associated with a higher prevalence of thyroid dysfunction. If we focused only on the UP level to avoid the effect of kidney disfunction itself, patients in category A3, i.e., those with total proteinuria of ≥0.50 g/day, showed the highest prevalence of OH and SH.

### Association of OH, SH, LT3S and LT4S with NS

The characteristics of the NS (nephrotic syndrome) and NNS (non-nephrotic syndrome) groups are presented in Table 3. Age and sAlb levels were significantly different between these two groups; however, sex and eGFR were not (Table 3a). sFT3 levels were significantly lower in the NS group; however, intergroup differences in sFT4 levels were not significant, and sTSH levels was significantly higher in the NS group (Table 3b). The OH and SH prevalence tended to increase in NS patients. However, we could not observe any tendencies for LT3S and LT4S in NS. Significantly fewer NS patients has normal thyroid function. In other words, laboratory testing consistent with hypothyroidism was predominantly found in nephrotic patients.

**Table 2b.** Thyroid hormones and binding proteins.

Parameter	Median (IQR)
sFT3 pg/ml	2.58 (2.09–2.96)
sFT4 ng/dl	1.14 (0.96–1.29)
sTSH μIU/ml	3.67 (2.0–7.41)
UT3 ng/ml	1.18 (0.033–0.818)
UT4 μg/dl	1.03 (0.706–1.49)
UTSH μIU/ml	0.119 (0.033–0.818)
UAlb μg/ml	735.9 (100.2–1642.7)
UpreAlb mg/dl	9.05 (4.3–14.025)
UTBG μg/ml	7.95 (5.5–14.45)

Abbreviations: sFT3: serum free T3; sFT4: serum free T4; sTSH: serum TSH; UT3: urine total T3; UT4: urine total T4; UAlb: urine albumin; UpreAlb: urine prealbumin; UTBG: urine thyroid-binding globulin; IQR: interquartile range.

Reference values: sFT3, 2.26–4.15 pg/ml; sFT4, 1.01–1.67 ng/dl; sTSH, 0.32–4.12 μIU/ml. There are no reference values for urinary data.

**Table 3a.** Characteristics of NS and NNS patients (*n* = 21 and 78, respectively).

Parameter		NS Median (IQR)	NNS Median (IQR)	
Age		5 (11–68.5)	64.5 (46–75)	*p* < 0.0265
Sex, male, n (%)		11 (52.45%)	40 (50.0%)	no gnificant differences (ns)
eGFR ml/min/1.73m^2^		14.6 (7.85–41.65)	23.1 (10.1–44.25)	Ns
Alb g/dl		2.1 (1.7–55)	3.9 (3.5–4.1)	*p* < 0.0001
CKD stage, %	1	9.5%	1.3%	
	2	9.5%	11.7%	
	3	14.3%	28.6%	
	4	14.3%	13%	
	5	52.4%	45.4%	R^2^ 0.0177
Thyroid function, %	OH	28.6%	17.9%	Ns
	SH	33.3%	19.2%	Ns
	LT3S	19%	15.4%	Ns
	LT4S	9.5%	10.3%	Ns
	Normal	9.5%	37.2%	*p* = 0.018

Abbreviations: NS: nephrotic syndrome; NNS: non-nephrotic syndrome; OH: overt hypothyroidism; SH: subclinical hypothyroidism; LT3S: low T3 syndrome; LT4S: low T4 syndrome; eGFR: estimated glomerular flow rate; Alb: albumin; IQR: interquartile range.

Reference values: eGFR > 60 mL/min/1.73m^2^, Alb≧3.9g/dl.

**Table 3b.** Characteristics of NS and NNS patients (*n* = 21 and 78, respectively).

Parameter	NS Median (IQR)	NNS Median (IQR)	
sFT3 pg/ml	2.13 (1.725–2083)	2.65 (2.168–2.97)	*p* = 0.0379
sFT4 ng/dl	1.07 (0.92–1.32)	1.15 (0.975–1.28)	Ns
sTSH μIU/ml	6.09 (3.365–11.675)	3.28 (1.898–5.913)	*p* = 0.0324
UT3 ng/ml	1.45 (0.9825–2.93)	1.18 (0.922–1.688)	Ns
UT4 μg/dl	2.22 (1.11–4.4)	0.9185 (0.6995-1.123)	*p* < 0.0001
UTSH μIU/ml	2.5 (0.2505–5.065)	0.0675 (0.032–0.3363)	*p* = 0.0002
UAlb μg//ml	3157.9 (1516.8–7103.2)	496.35 (59.925–1051.1)	*p* < 0.0001
UpreAlb mg/dl	9.05 (4.3–14.025)	0 (0)	*p* < 0.0001
UTBG μg//ml	8.05 (5.3–15.05)	7.7 (7.4–8.8)	Ns

Abbreviations: sFT3: serum free T3; sFT4: serum free T4; sTSH: serum TSH; UT3: urine total T3; UT4: urine total T4; UAlb: urine albumin; UpreAlb: urine prealbumin; UTBG: urine thyroid-binding globulin; IQR: interquartile range.

Reference values: sFT3, 2.26–4.15 pg/ml; sFT4, 1.01–1.67 ng/dl; sTSH, 0.32–4.12 μIU/ml.

Regarding excretion in the urine, UT4, UTSH, UAlb, and UpreAlb levels were significantly higher in the NS group; however, intergroup differences in UT3 and TBG levels were not significant (Table 3b).

**Figure 4.**
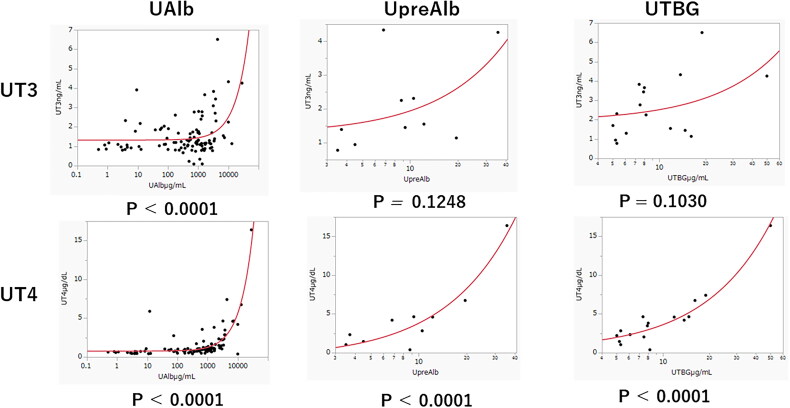
Correlation between thyroid hormones and hormone-binding proteins excreted in the urine. Abbreviations: UT3: urine total T3; UT4: urine total T4; UAlb: urine albumin; UpreAlb: urine prealbumin; UTBG: urine thyroid-binding globulin

The correlation between urine hormone levels and urine binding proteins is shown in Figure 4. UT3 was positively correlated with UAlb but not with UpreAlb or UTBG. In contrast, UT4 was positively correlated with all urine hormone-binding proteins, such as UAlb, UpreAlb, and UTBG.

### Antithyroid antibodies (ATAb); anti-thyroglobulin antibody (TgAb) and anti-thyroid peroxidase antibody (TPOAb)

Table 4 shows the number and percentage of TgAb and TPOAb. Of the 56 patients who underwent measurement of TgAb, 21 exhibited positive antibody (37.5%): normal (euthyroidism) 11 (19.6%), NTI 1 (1.8%), LT4S 1 (1.8%), OH 4 (7.1%), and SH 4 (7.1%). Likewise, 55 underwent measurement of TPOAb, 17 exhibited positive antibody (30.9%): normal (euthyroidism) 9 (16.4%), NTI 1 (1.8%), LT4S 1 (1.8%), OH 3 (5.5%) and SH 3 (5.5%). 35 exhibited negative TgAb (62.5%) including OH 8 (14.3%) and SH 9 (16.1%). 38 exhibited negative TPOAb including OH 9 (16.4%) and SH 9 (16.4%).

**Table 4.** TgAb and TPOAb.

	OH n, %	SH n, %	NTI n, %	LT4S n, %	Euthyroid n, %	Total n, %
TgAb positive	4, 7.1	4, 7.1	1, 1.8	1, 1.8	11, 19.6	21, 37.5
negative	8, 14.3	9, 16.1	7, 12.5	4, 7.1	7, 12.5	35, 62.5
TPOAb positive	3, 5.5	3, 5.5	1, 1.8	1, 1.8	9, 16.4	17, 30.9
negative	9, 16.4	9, 16.4	7, 12.7	4, 7.3	9, 16.4	38, 69.1

Abbreviations: TgAb: anti-thyroglobulin antibody; TPOAb: anti-thyroid peroxidase antibody; OH: overt hypothyroidism; SH: subclinical hypothyroidism.

Reference values: TgAb ≦ 28, TPOAb ≦ 16: antibody-positive, Values are number and percentage.

Referring to OH and SH, there were numerically more antibodies negative patients than positive patients.

## Discussion

This study found a high prevalence of hypothyroidism among CKD patients with proteinuria, regardless of sex and thyroid antibodies, indicating a significant association between hypothyroidism and the presence of UP. Second, we recognized that UT4 and UTSH levels were obviously higher in patients with high UP levels, which might have led to more patients with hypothyroidism (OH and SH) being observed in the NS group. These results prove clearly that the excretion of thyroid hormones in the urine must be one of the factors contributing to hypothyroidism. Third, LT3S or LT4S as non-thyroidal illness syndromes might not be related to the UP level, and there may be fewer non-thyroidal illness syndromes in CKD patients with proteinuria than expected.

This study followed our previous research on the prevalence of hypothyroidism in Japanese CKD patients [1]. In addition, we found a high prevalence of hypothyroidism among CKD patients with proteinuria and that the prevalence of OH and SH was higher among patients with advanced CKD and high UP levels. These results prompted us to thoroughly investigate the mechanisms of hypothyroidism in CKD patients in terms of the excretion of thyroid hormones and hormone-binding proteins in urine. The results of this study on the prevalence of hypothyroidism do not contradict our previous results.

Many studies have investigated the relationship between hypothyroidism and NS [13, 14, 19, 20]. In particular, thyroid hormone replacement therapy is necessary in children with congenital NS or in adults with NS, because of the loss of thyroid hormones in the urine [19, 21]. However, a consensus has not yet been completely reached. In 2022, Fukata et al. reported four cases of hypothyroidism due to NS [22]. They reported that the presence of a reduced thyroid reserve may predispose NS patients to hypothyroidism caused by the loss of protein-bound thyroid hormones and concluded that further urine tests are required. The direction of Fukata’s study didn’t contradict Fonseca and Bevenga’s reports as previously mentioned.

What is particularly noteworthy in our study is that we focused on thyroid hormones and thyroid hormone-binding proteins excreted in the urine to prove the excretion of thyroid hormones as previous reports. We also revealed that patients of OH and SH in this situation, were predominantly antibodies negative, and antibody positivity must have no effect on hypothyroidism when thyroid hormones are excreted into the urine. We found that UT4 and UTSH levels were significantly higher in the NS patients than in the NNS patients (Table 3). This led us to conclude that NS complicated by hypothyroidism is mainly attributable to the loss of thyroid hormones through the urine. Figure 4 shows that UT4 is strongly correlated with all hormone-binding proteins in urine, while UT3 is correlated only with UAlb. Most of the T4 is bound to proteins and only 0.02–0.03% is free in the serum. On the other hand, the percentage of FT3 in serum is 0.2–0.3%, which is approximately 10-fold that of FT4, indicating that T4 binds more strongly to proteins. This might explain why UT3 correlated only with UAlb, while UT4 correlated with UAlb, UpreAlb, and UTBG (Figure 4).

Few patients in the NNS developed hypothyroidism, including OH and SH [23]; however, there must be an obvious tendency for higher prevalence of OH and SH in the NS group. Thus, there should be greater awareness about the possibility of hypothyroidism not only in NS patients but also in CKD patients with proteinuria. Moreover, it is clinically important that proteinuria, even if small amounts of proteins are detected in the urine, might be a factor causing hypothyroidism independent of thyroid autoantibodies, in other words, independent of autoimmune thyroid disease. Kinoshita et al. speculated that there is a large amount of transfer of thyroid hormones with the binding proteins from blood vessels to the urine as well as to the third space as edema or pleural effusion [23]. Their logic may support the evidence that although the patient’s condition does not fulfill the diagnostic criteria of NS, edema may still cause hypothyroidism by sequestering T4 in the tissue.

In contrast, LT3S and LT4S involve different from hypothyroidism [9–12]. The mechanisms underlying these syndromes have been debated for decades; however, no clear consensus has been established. In particular, CKD, a chronic illness, may often be complicated by NTI [9, 24, 25]. In general, chronic illness might result in the suppression of the hypothalamus-pituitary-thyroid axis, reduction of T4 conversion to T3 with increased plasma reverse T3 (rT3), and reduction in the T3 demand in the peripheral organs as a possible biological reaction. However, several studies investigating NTI have reported that rT3 does not increase in kidney dysfunction [9, 26]. Patients with CKD are chronically ill and tend to be malnourished. These conditions would result in LT3 syndrome or even further deterioration, and we speculate that more patients might develop LT4 syndrome. In 2016, Fan et al. reported that 5.4% of non-dialysis CKD patients (*n* = 279) developed LT4 syndrome and 47% developed LT3S [24]. They concluded that the increasing prevalence of LT3 syndrome may be a predictor of worsening CKD, and LT3 syndrome is closely associated with malnutrition.

In contrast, considering the reference values, we found that the median sFT3 and sFT4 levels were normal in both the NS and NNS groups, and only sTSH levels were higher in the NS group (Table 3b). Although sFT3 and sFT4 levels are within the normal range, the sensor of TSH secretion in the pituitary must detect delicate changes as the secretion of T3 and T4 decreases. Consequently, a high level of sTSH must be a positive feedback mechanism in cases of NS and NNS, which may indicate that there may be fewer non-thyroidal illness syndromes in NS and NNS.

The NS and NSS groups did not significantly differ in terms of the prevalence of or tendency for LT3S. The same was true in the case of LT4S. These results strongly suggest that the factors causing LT3S and LT4S are unrelated to UP itself. There were not as many patients with LT3S or LT4S as we expected in those situations, as mentioned above. Considering these results, clinicians must pay more attention to making proper diagnoses for LT3S and LT4S, otherwise OH or SH who need thyroid hormone replacement therapy may be misdiagnosed as LT3S or LT4S. There is another possible explanation for this finding. Kaptein et al. observed reduced rT3 production rates in the presence of normal sFT4 levels, suggesting impaired conversion of T4 to rT3 [27]. This strongly supports our finding that sFT4 levels remained normal in both the NS and NNS groups. According to a recent report by Reinhardt [28], the concentration of rT3 correlated negatively with albuminuria and was significantly lower than that in patients with severe albuminuria. This means T3 production might not be interrupted in NS patients, but still a large amount of sFT3 bound to UAlb must be excreted into the urine in the NS patients (Figure 4).

sFT4 levels showed no difference between the NS and NNS groups (Table 3b). Increased secretion of sTSH by positive feedback may compensate enough T4.

In addition, the excretory disorder of iodine itself caused by kidney dysfunction should not be ignored, as excessive iodine can cause temporary hypothyroidism in CKD patients [29, 30]. However, it was difficult to determine which cases were affected by excess iodine in our study.

## Limitation

We did not measure the concentration of serum TBG in this study; consequently, we could only conclude that UTBG levels were not different between the NS and NNS groups. In addition, we did not measure Tg and TPO antibodies routinely in all subjects, but in 56 and 55 respectively. In reference to statistics, the methods had lack of multivariate analysis and we didn’t run Spearman rank coefficient non-parametric variables.

## Conclusion

In this study, we demonstrated a significant association between hypothyroidism and NS regardless of sex and autoimmunity. Urinary loss of thyroid hormones must be one of the factors of hypothyroidism independent of autoimmunity, and there were fewer patients with a non-thyroidal illness than we predicted among CDK patients with proteinuria. These results led us to confirm a proof-of-concept of nephrogenic hypothyroidism.
